# Pulmonary cryptococcosis characteristics in immunocompetent patients—A 20‐year clinical retrospective analysis in China

**DOI:** 10.1111/myc.12966

**Published:** 2019-08-04

**Authors:** Xiaomeng Hou, Lei Kou, Xiaozhen Han, Rui Zhu, Lan Song, Tao Liu

**Affiliations:** ^1^ Department of Pulmonary Medicine, Peking Union Medical College Hospital Peking Union Medical College and Chinese Academy of Medical Sciences Beijing China; ^2^ Department of Quantitative Health Sciences Cleveland Clinic Cleveland Ohio; ^3^ Department of Medical Record, Peking Union Medical College Hospital Peking Union Medical College and Chinese Academy of Medical Sciences Beijing China; ^4^ Department of Radiology, Peking Union Medical College Hospital Peking Union Medical College and Chinese Academy of Medical Sciences Beijing China

**Keywords:** China, clinical characteristics, Cryptococcosis, immune status, lung, nutrition immunity, radiograph distribution, retrospective

## Abstract

**Background:**

Pulmonary cryptococcosis (PC) is not considered an rare, opportunistic infection anymore. The immunocompetent population accounts for an increasing proportion of the morbidity.

**Objective:**

This study investigated the clinical characteristics of PC patients spanning 20 years, in a referral centre of China.

**Patients/Methods:**

We retrospectively investigated the clinical data of 99 patients with PC who were diagnosed at Peking Union Medical College Hospital (PUMCH) from January 1998 to December 2017.

**Results:**

Pulmonary cryptococcosis incidence in PUMCH has seen sharp increase in two decades. There were 40.4% (40/99), 17.2% (17/99) and 42.4% (42/99) immunocompetent, mildly immunocompromised and severe immunocompromised patients, respectively. Significantly higher (*P* = .035) male predominance in immunocompetent and mildly immunocompromised groups (68.4%, 39/57) compared with severe immunocompromised group (45.2%, 19/42) was found. Overall, 27.5% (11/40) immunocompetent patients reported a significant difference (*P* = .02) in history of more than weekly drinking, higher than mildly or severe immunocompromised. No significant difference occurred in symptoms and radiographic characteristics among the groups. In pulmonary computerised tomography findings, the non‐air pathway feature was the dominant distribution characteristics in all patients with PC (*P* = .002). The gap in body dissemination frequency between immunocompetent combined with mildly immunocompromised (5.26%, 3/57) and severe immunocompromised (19.0%, 8/42) was marginally significant (*P* = .05).

**Conclusions:**

Gender and alcohol drinking could be PC risk factors of concern in patients without severe immunodeficiency. No significant difference occurred in symptoms or radiographic characteristics between patients with different levels of immune status. The unique radiographic non‐air pathway distribution in the lung may be the feature of *Cryptococcus* invasion that may enhance accurate diagnosis.

## 1 INTRODUCTION

The most common clinical manifestation of Cryptococcosis is the pulmonary system infection.^1^ However, *Cryptococcus* also has a propensity to infect the central nervous system (CNS).^2^, ^3^ The innate mechanism of pulmonary cryptococcosis (PC) is that of an opportunistic fungal infection that mostly affects immunocompromised patients, especially patients with human immunodeficiency virus (HIV). The most common means by which *Cryptococcus* causes infection in the lungs is via inhalation.^1^ Recently, a retrospective review of non‐AIDS patients pathologically diagnosed with PC from China revealed that 60% of PC cases were diagnosed in immunocompetent non‐HIV patients.^4^ The prevalence of cryptococcosis in non‐HIV patients especially in non‐HIV non‐immunocompromised patients and the mechanism of the interaction between host and pathogen are being given increasing attention.^5^, ^6^

Pulmonary cryptococcosis is easily misdiagnosed as lung cancer, pulmonary tuberculosis and other pulmonary mycoses, both clinically and radiologically.^1^ Misdiagnosis and underdiagnosis is particularly common in patients with normal immunity. The clinical characteristics and the possible pathophysiologic mechanism of PC in immunocompetent individuals may become increasingly important for an accurate diagnosis. Therefore, this study was aimed to investigate the clinical characteristics of PC patients spanning 20 years, in a single centre of China.

## 2 PATIENTS AND METHODS

We retrospectively reviewed the medical records of 116 hospitalised patients who were diagnosed with ‘PC’ from January 1998 to December 2017 in Peking Union Medical College Hospital (PUMCH), the most authoritative referral centre in the Chinese hospital rankings of Fudan University.

### 2.1 Ethics statement

The study was approved by the Ethical Committee of PUMCH (protocol number: s‐k 206). Because of the retrospective nature of the study, informed consent was waived. The patients' clinical characteristics data were reviewed and re‐evaluated by our research team including two senior pulmonologists and one radiologist.

### 2.2 Diagnostic criteria

The diagnosis of PC is usually based on a combination of clinical and radiological suspicion and laboratory confirmation.^7^ Of the 116 patients reviewed, 99 met the deterministic diagnostic requirements for PC (a case was excluded if such did not meet any one out of the four diagnostic requirements). These requirements were as follows:

1. Appropriate clinical and radiographic evidence of active pulmonary infection with exclusion of other pulmonary pathogens that might explain the present clinical course, such as mycobacteria.
2. At least one positive result of the following tests:
  - Confirmed pathological evidence of the organism from tissue samples obtained by transbronchial biopsy, computerised tomography (CT) guided percutaneous biopsy, open biopsy or resection of the lung.
  - A positive culture of *Cryptococcus* from bronchoalveolar lavage, pleural fluid, cerebrospinal fluid or from blood samples.
  - Positive serum cryptococcal antigen test.^8^, ^9^, ^10^
3. Exclusion of any case with a clear evidence of combined infection of the lung with other pathogens.
4. Exclusion of other lung diseases that may explain the current clinical manifestations such as lung cancer.

### 2.3 Predisposing conditions

Immunocompromised conditions such as HIV infection, organ transplantation, diabetes mellitus, corticosteroid or immunosuppressive therapy, and malignancy are linked with PC.^11^, ^12^

After extensive review of the clinical data, all diagnosed 99 cases were classified into three categories by their predisposing conditions:

1. We considered patients who had major discernible immunodeficiency (such as HIV infection, haematologic malignancy) or showed a certain degree of immunocompromise due to immunosuppressive therapy (such as organ transplantation, malignancy under radiation therapy or chemical therapy, or Cushing syndrome); as well as patients who were under active immunosuppressive therapy due to their autoimmune or inflammatory disease, to be severe immunocompromised.
2. We considered patients who had relevant systematic diseases such as diabetes mellitus, chronic liver disease and other endocrine diseases like hypothyroidism that may affect immune status to be mildly immunocompromised.
3. Finally, we considered other patients who had no relevant systematic diseases or a history of certain immunocompromised, to be immunocompetent.

A single patient with one or more of the above conditions was classified by the most primary predisposing condition according to their immunity.

### 2.4 Statistics

Descriptive statistics were used to characterise patient cohorts. Mean (standard deviation [SD]) was used to summarise continuous variables; frequency (1st Quartile, 3rd Quartile) was used to summarise categorical variables. *T* test was used to compare continuous variables across groups, while Chi‐squared test and Fisher's exact test were used to compare categorical variables. Statistical significance was established with two‐sided *P* values <.05. All analyses were conducted in R (3.5.2; Foundation for Statistical Computing).

## 3 RESULTS

### 3.1 Annual incidence

**Figure 1:**
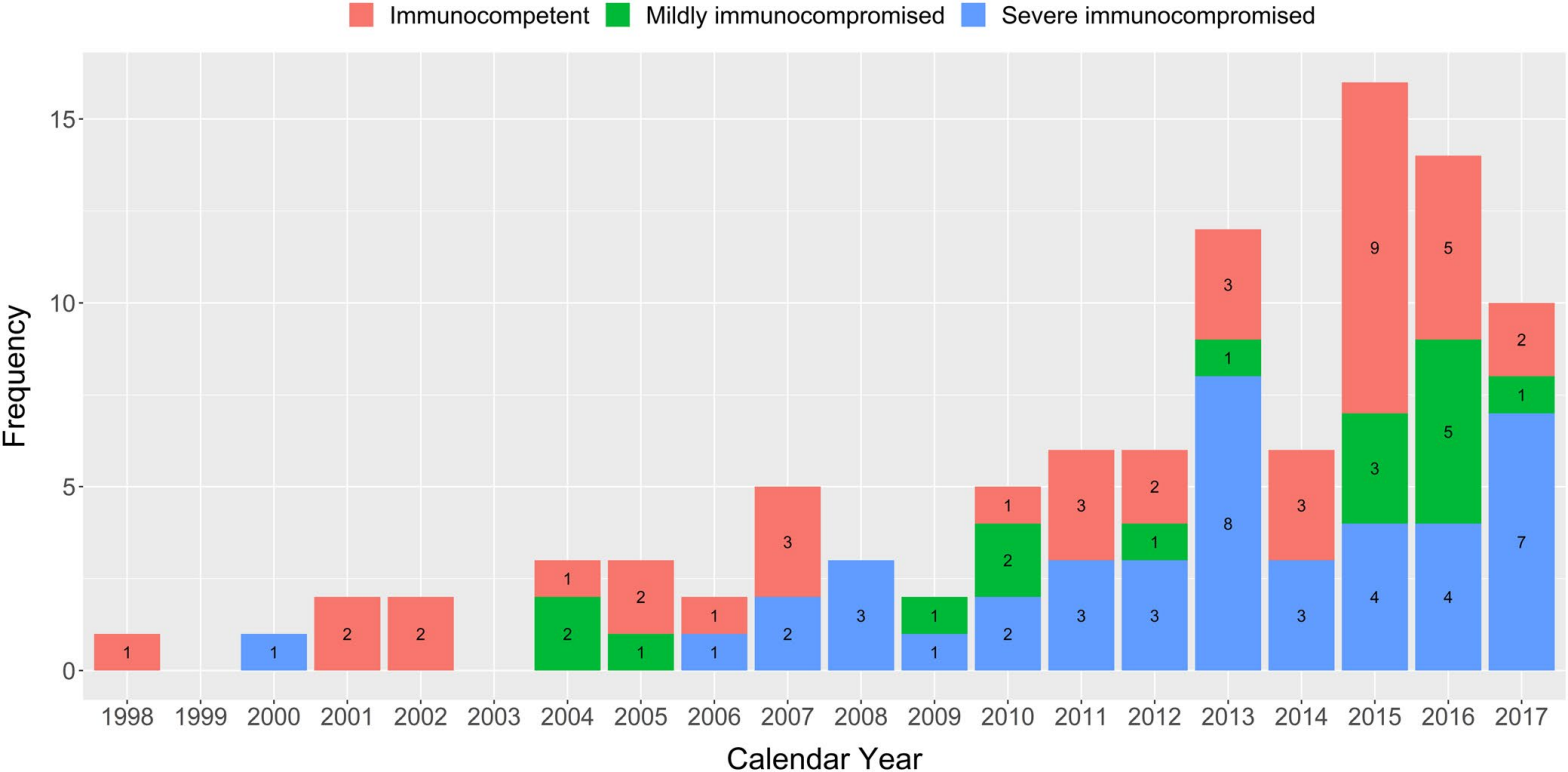
Annual incidence

A total of 99 patients with PC were included in this study. As shown in Figure 1, there were only six cases diagnosed between 1998 and 2002, while 58 cases were diagnosed from 2013 to 2017. The incidence by groups of mildly immunocompromised, severe immunocompromised and immunocompetent groups is also shown in Figure 1.

### 3.2 Predisposing conditions

Among these 99 patients, 40.4% (40/99) were immunocompetent, 17.2% (17/99) were mildly immunocompromised, and 42.4% (42/99) were severe immunocompromised (Table 1).

**Table 1:** Predisposing conditions in the 99 PC patients

Underlying disease	n	Percentage
Immunocompetent	40	40.4
Mildly immunocompromised	17	17.2
Diabetes	11	11.1
Chronic liver disease	2	2.0
Chronic renal failure	1	1.0
Puerperium	1	1.0
Primary hyperaldosteronism	1	1.0
Hypothyroidism	1	1.0
Severe immunocompromised	42	42.4
AIDS	2	2.0
Common variable immunodeficiency disease	1	1.0
Cushing syndrome	8	8.1
Solid‐organ malignancy in chemotherapy	8	8.1
Autoimmune or inflammatory disease under continuous corticosteroid or immunosuppressive therapy	17	17.2
Haematologic malignancy	5	5.1
Heart transplantation	1	1.0

Abbreviations: AIDS, acquired immunodeficiency syndrome; PC, pulmonary cryptococcosis.

### 3.3 Demographic characteristics of patients with PC

The demographic characteristics of all 99 PC patients are shown in Table 2. The ratio of male to female was 1.4:1 (58:41). In mildly immunocompromised and immunocompetent groups, PC infection was more frequent in male (68.4%, 39/57, *P* = .035) compared to the severe immunocompromised group. Patients age ranged from 18 to 83 years, and the mean age was 49.3 (14.3) years. Ever smokers accounted for 22.2% (22/99), and 15.2% (15/99) were drinkers (drinking weekly to daily).

**Table 2:** Demographic characteristics of PC patients by immune status

	Immunocompetent	Mildly immunocompromised	Severe immunocompromised	Overall	*P*
	N = 40	N = 17	N = 42	N = 99	
Age (y, SD)	48.5 (11.1)	54.8 (13.6)	47.8 (16.9)	49.3 (14.3)	.214
Gender					
Male	27 (67.5%)	12 (70.6%)	19 (45.2%)	58 (58.6%)	.067
Male	39 (68.4%)		19 (45.2%)		.035
Smoking					
Ever	6 (15.0%)	5 (29.4%)	11 (26.2%)	22 (22.2%)	.328
Drinking					
Seldom	29 (72.5%)	16 (94.1%)	39 (92.9%)	84 (84.8%)	
Often (more than weekly)	11 (27.5%)	1 (5.9%)	3 (7.1%)	15 (15.2%)	.02
Exposure history					
Positive	12 (30.0%)	5 (29.4%)	5 (11.9%)	22 (22.2%)	.091

Abbreviations: PC, pulmonary cryptococcosis; SD, standard deviation.

By environmental factors, 22.2% (22/99) had exposure to environmental risk factors including close, frequent and direct exposure to pigeon or poultry droppings or had ever had a period in humid working environment. The comparison of these demographic characteristics was by the three groups (mildly immunocompromised, immunocompetent and severe immunocompromised) is shown in Table 2.

### 3.4 Clinical manifestations of PC

#### 3.4.1 Symptoms

In the 99 PC patients, the most common symptoms were cough (55.6%, 55/99), sputum (37.4%, 37/99), chest pain (8.1%, 8/99), fever (25.3%, 25/99) and asymptomatic (28.3%, 28/99). The comparison between the three immune status groups is shown in Table 3.

**Table 3:** Symptoms in PC patients by immune status

	Immunocompetent	Mildly immunocompromised	Severe immunocompromised	Overall	*P*
	N = 40	N = 17	N = 42	N = 99	
Cough	26 (65.0%)	9 (52.9%)	20 (47.6%)	55 (55.6%)	.278
Sputum	16 (40.0%)	6 (35.3%)	15 (35.7%)	37 (37.4%)	.905
Chest pain	5 (12.5%)	2 (11.8%)	1 (2.4%)	8 (8.1%)	.169
Fever	6 (15.0%)	4 (23.5%)	15 (35.7%)	25 (25.3%)	.108
Hemoptysis	3 (7.5%)	1 (5.9%)	2 (4.8%)	6 (6.1%)	.864
Dyspnoea	3 (7.5%)	2 (11.8%)	7 (16.7%)	12 (12.1%)	.504
Asymptomatic	11 (27.5%)	5 (29.4%)	12 (28.6%)	28 (28.3%)	>.99

Abbreviation: PC, pulmonary cryptococcosis.

#### 3.4.2 Radiographic feature

**Figure 2:**
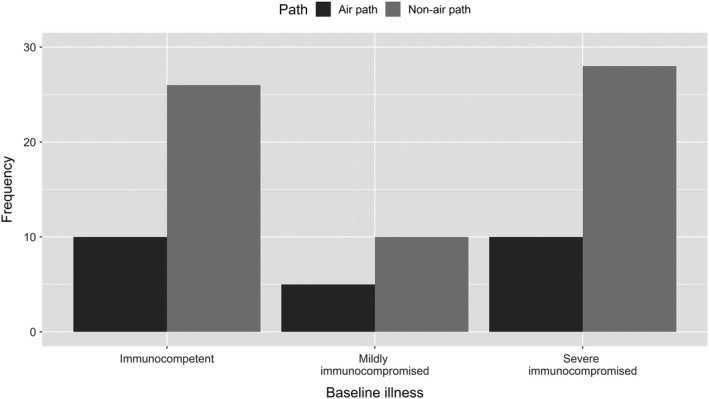
The features of distribution of PC lung lesions in CT imaging. CT, computerised tomography; PC, pulmonary cryptococcosis

The most common chest CT findings among the 99 patients were pulmonary nodule (74.7%, 74/99), infiltration (40.4%, 40/99) and cavitation (24.2%, 24/99). Most of the shadows (71.7%, 71/99) showed multiple modes. The comparison of common chest CT findings among the three immune status groups showed no significant difference, Table 4. Different from previous studies, we focused on the distribution of lung lesions on CT imaging. The right side of the lung and involvement of the upper lung bilaterally, with the characteristics of air‐inhalation mechanism, were classified as air path type, while other distributions including double lower, double scattered and left lower were classified as non‐air path type. Our data showed significant priority of non‐air path type distribution (*P* = .002) according to the air path type in all patients, Table 4, Figure 2.

**Table 4:** Radiographic features in PC patients by immune status

	Immunocompetent	Mildly immunocompromised	Severe immunocompromised	Total	*P* overall
	N = 40	N = 17	N = 42	N = 99	
Nodule	29 (72.5%)	13 (76.5%)	32 (76.2%)	74 (74.7%)	.953
Infiltrates	17 (42.5%)	7 (41.2%)	16 (38.1%)	40 (40.4%)	.918
Cavity	8 (20.0%)	6 (35.3%)	10 (23.8%)	24 (24.2%)	.499
Mediastinal lymph node enlargement	3 (7.50%)	2 (11.8%)	5 (11.9%)	10 (10.1%)	.746
Distribution frequency					
Single	11 (27.5%)	5 (29.4%)	9 (21.4%)	25 (25.3%)	.485
Multiple	29 (72.5%)	12 (70.6%)	30 (71.4%)	71 (71.7%)	
Diffused	0 (0.00%)	0 (0.00%)	3 (7.14%)	3 (3.03%)	
Location					
Double lower	7 (19.4%)	3 (20.0%)	3 (7.89%)	13 (14.6%)	.931
Double scattered	12 (33.3%)	5 (33.3%)	17 (44.7%)	34 (38.2%)	
Left lower	7 (19.4%)	2 (13.3%)	8 (21.1%)	17 (19.1%)	
Left upper	4 (11.1%)	2 (13.3%)	3 (7.89%)	9 (10.1%)	
Right lower	5 (13.9%)	3 (20.0%)	6 (15.8%)	14 (15.7%)	
Right upper	1 (2.78%)	0 (0.00%)	1 (2.63%)	2 (2.25%)	
Distribution					
Air path	10 (27.8%)	5 (33.3%)	10 (26.3%)	25 (28.1%)	.904
Non‐air path	26 (72.2%)	10 (66.7%)	28 (73.7%)	64 (71.9%)	
				*P* = .002	

Abbreviation: PC, pulmonary cryptococcosis.

#### 3.4.3 Dissemination

The frequency of body dissemination of cryptococcus showed significant difference between the three immune status groups (Table 5).

**Table 5:** Infection *dissemination frequency* in PC patients by immune status

	Immunocompetent	Mildly immunocompromised	Severe immunocompromised	Overall	*P*
	N = 40	N = 17	N = 42	N = 99	
Disseminate					
Positive	2 (5.00%)	1 (5.88%)	8 (19.0%)	11 (11.1%)	.106
Positive	3 (5.26%)		8 (19.0%)		.05

Abbreviation: PC, pulmonary cryptococcosis.

## 4 DISCUSSION

Cryptococcosis remains the second most common cause of AIDS‐related mortality.^13^ Increasing incidence of PC in China has been reported in recent decades.^14^ The incidence of PC in PUMCH has seen a 10 times increase in the past two decades in our data. On the one hand, the prevalence of the disease itself increased with the amount of the patients with iatrogenic immunosuppression and the survival of the immunosuppressed patients. On the other hand, the widespread application of serological tests and chest CT, video‐assisted thoracoscopic surgery, transbronchial lung biopsy and CT‐guided percutaneous lung puncture techniques also contribute greatly to the increase. In addition, the better awareness of this disease by physicians is also attributed to this increasing trend.^13^, ^15^, ^16^

Cryptococcosis used to be a rare opportunistic infection that commonly affects the lungs. Individuals with compromised cellular immunity are at higher risk and were typically patients with AIDS.^17^, ^18^ In China and Japan, cryptococcosis infection is prevalent predominantly in HIV‐negative populations.^6^, ^19^ These were patients who received prolonged treatments with corticosteroids and/or immunosuppressive agents, who received solid organ and hematopoietic stem cell transplantation, or who had haematologic and solid organ malignancies.^17^, ^18^, ^20^

Although it is much less common, individuals with a milder degree of immune suppression (such as splenectomy, cirrhosis, diabetes mellitus and pregnancy) have an increased risk of *Cryptococcus* infections.^21^, ^22^ In our study, patients with diabetes, as the primary underlying disease, accounted for 11.1% (11/99). Hyperglycaemia in patients with diabetes could have led to decreased cell‐mediated immunity that could explain the association between diabetes and cryptococcosis.^23^ The relationship between other systemic diseases and abnormal immunity remains to be further studied.

Recently, a retrospective review in China revealed that 60% of PC cases were diagnosed in immunocompetent non‐HIV patients^4^ and another study reported that 67% of PC in immunocompetent patients were disseminated into the CNS.^24^ The prevalence of cryptococcosis in non‐HIV patients especially in non‐HIV non‐immunocompromised patients is being given increasing attention. In our study, 40.4% (40/99) overall were immunocompetent. Research has found that *Cryptococcus neoformans* can be found globally, and that it predominantly affects the immunosuppressed, whereas *Cryptococcus gattii* is endemic to certain regions and often affects immune‐competent individuals.^25^, ^26^ PC infection is more frequent in male than female.^27^, ^28^ The gender difference in host immune systems is a possible explanation for the male predominance among patients with cryptococcosis.^29^ Our study showed significantly higher male gender predominance in immunocompetent and mildly immunocompromised groups compared with severe immunocompromised group, which also support this previous finding. Overall, 27.5% (11/40) of our immunocompetent patients reported the history of more than weekly drinking, which was significantly higher than other immunocompromised patients, 5.9% (1/17) in mildly immunocompromised and 7.1% (3/42) in severe immunocompromised. Interestingly, it was also much higher than the prevalence of alcohol use disorder (AUD) in the general population (10.1%) in mainland China.^30^ However, we were unable to conduct a comprehensive data collection according to AUD diagnostic criteria. Chronic alcohol drinking reduces the level of antioxidant, glutathione, within the alveolar spaces, decreases the production of alveolar epithelial surfactant, impairs airway barrier integrity, decreases alveolar macrophage engulfment and renders the lung susceptible to pathogenic infections.^31^, ^32^ In addition, A few studies reported the genetic predisposition to cryptococcosis in the Chinese population.^33^, ^34^, ^35^ All of the above factors may be associated with *Cryptococcus* infection in people with normal immunity.

Both innate and adaptive immune systems are involved in host response against *Cryptococcus* infection.^1^ How the different aspects of the host immune system protect against cryptococcal infection is highly complex. Our understanding of normal immunity and the defects in immunocompromised are still incomplete.^5^

In our study, the most common symptoms reported were cough (55.6%, 55/99), sputum (37.4%, 37/99), fever (25.3%, 25/99) and asymptomatic (28.3%, 28/99). Several reports demonstrated that the symptoms in immunocompetent PC patients were mild or asymptomatic.^6^, ^16^, ^19^, ^36^ According to our data, all symptoms did not differ significantly between groups by immune status.

The most common findings presented on chest CT were nodules (74.7%, 74/99), infiltrates or consolidation (40.4%, 40/99) and cavities (24.2%, 24/99). All the radiographic features did not differ significantly among our groups by immune status. In the literature, nodules with typical findings on CT scan, and cavitation of the nodules and/or masses have been reported to be more common in immunocompromised patients.^16^, ^27^

Independent of the type of cryptococcal infection or the primary organ involved, the fungus typically enters the body through the lungs.^37^ Unlike previous studies, we focused on the distribution of lung lesions on CT imaging, which also suggests the route for pathogens entry into the body. From common knowledge, air‐inhalation infections (air path) could have priority to attack the right or both upper lungs. However, our data on the sites invaded by *Cryptococcus* showed a significant priority distribution type for both lungs and the lower lung (*P* = .002). The study on the interaction mechanism between *Cryptococcus* and its host suggests that the yeast or spores of *Cryptococcus* could be escaping the respiratory defenses before reaching the distal lung; nutrition and temperature at the site of contact play a key role in germination.^5^, ^38^ The characteristics of the distribution of the lesions in our study might have been the result of the selected nutritional status and temperature by the fungal spores.

Ease of escape from the lungs and dissemination throughout the host is another distinctive feature of *Cryptococcus*. *Cryptococcus* can invade the bone, skin, eyes and the prostate, and may even cause disseminated disease and cryptococcemia. These presentations can occur in both immunosuppressed and immunocompetent individuals.^25^ Some studies suggest that cryptococcal meningitis was less frequent in HIV‐negative patients than patients with AIDS.^39^ Our study also found the frequency gap in the dissemination between immunocompetent group combined with mildly immunocompromised group (5.26%, 3/57) and severe immunocompromised group (19.0%, 8/42), which was significant (*P* = .05). A limitation to this study was the retrospective study design, which is limited by the integrity of the medical records.

## 5 CONCLUSIONS

Our finding suggests that gender and alcohol drinking could be PC risk factors of concern in patients without severe immunodeficiency. It is worth mentioning that in terms of the distribution of invading lung lesions, most of the patients had unique non‐air path inhalation contact distribution. The possibility of lung cryptococcus infection should be considered when the patient has lung lesions with the special non‐air path inhalation contact distribution pattern. Also, the mechanism of this special distribution pattern needs to be further studied according to the nutrient environment and temperature in the lung.

## CONFLICT OF INTEREST

There was no conflict of interest influenced the author's objectivity.

## AUTHOR CONTRIBUTIONS

Xiaomeng Hou conceived the ideas; Xiaomeng Hou, Rui Zhu and Lan Song collected the data; Lei Kou and Xiaozhen Han analysed the data; Xiaomeng Hou and Tao Liu led the writing.
